# The costs of participation in and delivery of community sport in Australia—a narrative review

**DOI:** 10.3389/fspor.2025.1641527

**Published:** 2025-09-23

**Authors:** Hans Westerbeek, Rochelle Eime, Katherine Owen

**Affiliations:** ^1^ Institute for Sustainable Industries & Liveable Cities (ISILC), Victoria University Business School, Melbourne, VIC, Australia; ^2^ Federation University Australia Institute of Health and Wellbeing, Ballarat, VIC, Australia; ^3^ The University of Sydney Prevention Research Collaboration, Sydney, NSW, Australia

**Keywords:** cost of sport, costs of participation, costs of delivering sport, sport production, affordability of sport, costs of sport review

## Abstract

Rising financial costs are undermining equitable access to community sport and threatening the sustainability of grassroots delivery systems. This narrative review synthesises peer-reviewed and grey literature, focusing on the Australian context, to examine the costs of participating in and delivering community sport. Evidence confirms that affordability remains a persistent barrier across all age groups, disproportionately affecting low socio-economic populations, culturally diverse groups, Indigenous communities, and people with disabilities. In parallel, community sports clubs are challenged by escalating facility, insurance, and staffing costs, declining volunteer numbers, and uncertain revenues, resulting in increasing reliance on participant fees and short-term fundraising. Strategies to reduce financial barriers include voucher schemes, tax rebates, grant programs, and charity-based initiatives. While these interventions provide temporary relief, they often benefit higher-income families more and rarely achieve long-term participation sustainability. Comparative international insights highlight that structural differences in funding models, ranging from heavily subsidised European systems to pay-to-play models in the United States, shape affordability and access in distinct ways. Policy implications point to the need to reposition community sport as a public good embedded in preventive health and equity frameworks, rather than as a consumer service. Achieving inclusive and sustainable systems requires moving beyond universal supports toward equity-focused, co-designed solutions that target priority groups, enhance club capacity, and also recognise the role of informal sport in providing low-cost opportunities. Future research should evaluate the long-term impact of financial interventions, develop robust economic models of return on investment, and examine the potential of digital innovation to alleviate cost pressures.

## Introduction

Physical inactivity poses a significant public health challenge, contributing to numerous chronic diseases and escalating healthcare costs globally (1). Engagement in physical activity (PA) across domains such as transport, domestic chores, work, and leisure is essential for health promotion (2). Notably, participation in organised community sport during leisure-time offers a range of benefits, including enhanced physical health, mental wellbeing, and social connectedness (3).

However, the costs associated with playing community sport is a major barrier across all ages. Research indicates that cost is a main barrier for children and youth, with expenses related to registration fees, equipment, and travel, therefore limiting access, particularly for low-income families (4, 5). The AusPlay survey reported that cost is a barrier for 37% of girls compared with 20% of boys (6). While at face value the financial costs of participation may be assumed to be the same across genders, several factors explain why girls report cost as a greater barrier. Research has shown that girls are more likely to participate in sports that require higher ongoing financial commitments, such as dance, gymnastics, and equestrian activities, where expenses for coaching, uniforms, and competitions are considerable (7, 8). In addition, families may prioritise boys' sport participation when household budgets are constrained, reflecting enduring cultural norms and gendered expectations (9). A recent analysis of youth sport in Australia confirmed that girls from low socio-economic backgrounds are disproportionately excluded when fees, equipment, or travel costs increase, thereby compounding both gender and class-based inequities (10). These findings suggest that the reported “economic barrier” is not merely a matter of absolute cost, but rather the interaction of sport type, family priorities, and broader social structures that shape access to participation opportunities.

The recent rise in living costs has further intensified these financial challenges. Although peer-reviewed studies on this specific impact are limited, existing research suggests that increased financial pressures lead individuals to modify their physical activity behaviours, often reducing participation in organised sports (11). This trend disproportionately affects those from lower socioeconomic backgrounds, potentially widening existing disparities in health outcomes (11). Moreover, the financial strain extends to community sports organisations. Rising operational costs and declining revenues have led some local sports clubs to consider closure, threatening the sustainability of community-based sports programmes (12). This not only limits opportunities for participation but also diminishes the social cohesion and support networks that clubs can provide. Volunteer numbers have also been impacted, leaving remaining volunteers to bear a heavier workload, posing long-term challenges for retaining both the paid and unpaid workforce essential to the operation of these clubs (11).

Addressing the financial barriers to sport participation is crucial for promoting involvement in sport across the socio-economic spectrum and ensuring equitable access to the benefits of being active. Strategies such as implementing pricing strategies, providing subsidies or vouchers, and enhancing community support systems have shown promise in mitigating these barriers (5, 13). A comprehensive understanding of the costs associated with playing sport and effective interventions to reduce these costs is essential for fostering inclusive participation and improving health outcomes across diverse populations.

In this scoping paper, relevant insights from academic publications and industry reports, in particular in the Australian context, are reviewed. In the first part, the perspective of the sport participant, or player is provided. This is followed by insights about the costs of sport delivery by clubs. In the last part, various cost reduction strategies are reviewed, and how (various international) government policy initiatives are developed and implemented in regard to the cost of sport participation. Recent scholarship, in that regard, cautions against treating “formal” and “informal” sport as fixed categories. Instead, participation sits along a continuum of (in)formalisation, shaped by how spaces, institutions and practices are negotiated and governed. From this perspective, affordability and access are partly determined by the power relations that stabilise (or destabilise) resources and recognition across both club-based and self-organised settings. We draw on this perspective to interpret the cost findings in this review and to frame policy options that address not only prices and fees, but also the governance processes through which opportunities to participate are created or constrained (14). The paper concludes with suggested policy implications and proposed avenues for future research.

## Cost to play community sport for participants

Participation in community sport involves various costs, which have been increasing in recent years. The Australian Sports Commission (15) (ASC) reported that Australians spent $18.7 billion on sport and physical activity in the 2022–2023 financial year, a significant increase from $10.7 billion five years earlier. This rise underscores the growing financial commitment required for individuals and families to engage in sporting activities. Importantly, the cost to play sport differs markedly across nations and continents. In the United States, for example, most sport participation is privately organised through community competitions rather than being sanctioned by national sport federations. This often results in a pay-to-play model that places a substantial financial burden on families. By contrast, in Europe—particularly in Northern Europe—although participation is also largely club-based, as in Australia, governments heavily subsidise community sport. These subsidies not only cover the full funding of playing facilities but also provide significant support to sport governing bodies, many of which have limited access to commercial revenue. Such differences highlight the varying structural and financial models that shape access to sport globally.

The AusPlay survey, conducted by the Australian Sports Commission (16), offers insights into the financial aspects of sport participation. This survey also collects data on the proportion of individuals who pay to play sport and the amounts they pay annually to organisations in registration fees. However, it's important to note that these figures represent payments made directly to organisations that manage or deliver the sport, and do not encompass additional expenses such as equipment, travel, and uniforms. Moreover, the components included in the payments to organisations can vary across different sports; some may cover court or field fees and uniforms, while others may not (17). AusPlay data shows that the average annual expenditure per adult on sport and physical activity participation rose from AUD $796 in 2018–19 to AUD $1,304 in 2022–23, a nominal increase of 64% (18). After adjusting for cumulative inflation of approximately 16% across the same period, this still represents a real increase of 41%, with adults in 2022–23 effectively paying AUD $1,124 in 2018–19 dollars. For children, family expenditure nearly doubled from an estimated AUD $685 in 2018–19 to AUD $1,369 in 2022–23. Once adjusted for inflation, the effective 2018–19 dollar cost equates to AUD $1,180, indicating a real increase of 72%. These results confirm that the rising financial burden on households is not solely an effect of general price inflation, but reflects substantive cost pressures specific to organised community sport participation. It is crucial to acknowledge that these figures may mask significant variations in costs associated with different sports, age groups and level of competition. For instance, sports requiring specialised equipment or facilities, and/or professional coaching such as golf or equestrian activities, often entail higher participation costs compared to sports like running or soccer, which require minimal equipment. Additionally, competitive levels, frequency of participation, and geographic location can influence the overall cost to participants. Further, the costs for local community sport participation are considerably lower than for players at the state, national or international levels.

Understanding the comprehensive costs associated with community sport participation is essential for developing strategies to mitigate financial barriers and promote equitable access to sport for all. To that end it is also important to take a closer look at those who are at a disadvantage when it comes to playing community sport. This narrative review aims to summarise (a) the cost barrier across priority subgroups; (b) the costs to play community sport; (c) the cost to deliver community sport; (d) policies and strategies to improve the affordability of sport.

## Method

A narrative literature search was conducted using Google Scholar; Frontiers; SpringerLink and Taylor and Francis for the period 2015–2024, applying combinations (AND, OR) of the search terms such as: cost to play sport Australia; cost of sport; club sport cost participate; sport policy cost; cost to deliver sport for clubs; and organisational capacity sports clubs. For example, combinations like: cost AND sport OR club sport OR community sport OR sport participate OR deliver sport OR organisational capacity sport. To complement this, formal reports and data sets available through the Australian Sports Commission (19) (ASC) Clearinghouse for Sport were also reviewed, given their relevance to policy and practice. We acknowledge that such official reports may carry an inherent degree of institutional bias, particularly when commissioned by sport agencies with vested interests. Nonetheless, they constitute the most comprehensive, nationally representative, and publicly available sources of data on sport participation and expenditure in Australia and therefore were considered appropriate to include alongside peer-reviewed literature. The review applied the following inclusion and exclusion criteria: Included were English language publications; research and reports presenting financial data in AUD; costs related to direct sport participation expenses such as equipment, registration, uniforms, and travel. Excluded were studies focusing on COVID-19, professional or elite sport, sport-for-development programs, gambling, sports injuries, and clinical/medical studies. Costs expressed solely in terms of time investment (rather than financial outlay) were also excluded. This process identified both academic and grey literature relevant to the costs of sport participation and delivery, with particular attention to the Australian context. We note as a limitation that the number of items initially screened and excluded was not systematically recorded, which represents an oversight in the reporting of this narrative review. Future iterations of this work will adopt a more structured recording of search results to enhance transparency.

## Disadvantaged groups

Participation in sport is closely linked to socio-economic status (SES), with individuals from higher SES backgrounds typically engaged in sport at greater rates compared to those from lower SES backgrounds. A systematic review and meta-analysis of 40 studies found that children and adolescents from high SES backgrounds are 1.9 times more likely to participate in sport and for longer durations (20). However, this positive association does not apply uniformly across all sports and activities (21). For instance, an Australian study revealed that SES was positively associated with participation in most sports, especially in niche activities such as canoeing/kayaking and rock climbing, as well as indoor activities that require additional infrastructure, equipment, or commercial facilities. Participation in sports that require access to water or snow was also more prevalent among high SES groups. Conversely, individuals from low SES backgrounds were more likely to participate in certain mainstream sports like outdoor cricket, rugby league, and touch football. Sport participation is shaped by a complex interplay of individual SES factors, such as income and education, as well as area-level factors like access to and proximity of facilities (22).

Other disadvantaged groups when it comes to participation in community sport include migrant children and youth. Their opportunity to participate is often constrained by financial costs and other barriers specific to migrant communities, such as religious and culturally determined norms (23). The cost barrier is not always due to an inability to pay, but rather a matter of different priorities, such as sending children to religious schools or travelling to visit family overseas. Participation in organised sport may not be a cultural norm for families who previously engaged in free “street” sports in their home countries (3). Sometimes local sports clubs conduct internal fundraising to assist those players who want to play but cannot afford to (23). It is often the case that specific programs for refugee-background children and youth are designed to eliminate the cost barrier and are offered free of charge (23). However, continually being able to source funding from internal fundraising or applying to various other organisations remains a major barrier to the sustainability of opportunities to play (23). Programs are labour-intensive and often require direct outreach to parents to encourage their children's participation. Many of these programs aim to promote integration and inclusion for migrants, providing them with a gateway to Australian culture. However, migrant children may also face overt racism in mainstream sport settings, necessitating that organisations develop strategies to address such incidents. While cost is a common barrier, other unique challenges faced by different demographic groups must also be addressed.

Indigenous Australians are much less likely to play sport than non-Indigenous Australians (24–26). Indigenous Australians encounter cost, racism, and cultural mismatch, compounded by transport barriers in remote areas (24, 25). People living with disabilities also participate less in sport compared to those without disabilities, largely due to financial constraints (4, 27, 28). Additional costs for this group include specialised equipment, such as sport-specific wheelchairs, transport, and paying support workers to attend sport sessions. The recent increase in the cost of living in Australia has disproportionately affected people with disabilities, further limiting their ability to be active (29). While Australia's National Disability Insurance Scheme provides funding to help individuals with disabilities access services, including sport, many encounter challenges with the scheme, such as inconsistent information and complex administrative processes.

## Who pays to participate in organised sport

Financial commitment to organised community sport varies notably between children and adults, with a higher proportion of children requiring payments for participation. According to AusPlay data, approximately 96% of children engaged in organised sport necessitated a payment, a trend that has remained consistent over time. This high percentage reflects the structured nature of children's sports, which are predominantly facilitated through local sports clubs or associations where membership fees are customary (30).

In contrast, the proportion of adults who pay to participate in sport is lower and decreases with age. Data from the 2023–2024 period indicates that 70%–73% of individuals aged 15–34 years paid to participate, compared to 63% of those aged 35–44 years, 53% of those aged 55–64 years, and just 45% of those aged 65 years and above (31). This trend aligns with previous research demonstrating that adults engage in sport across various settings, including community groups and workplaces, many of which do not require payment for participation (30). Additionally, adults often partake in informal or self-organised activities, such as running or casual team sports, which may not involve formal organisational fees. Understanding these participation patterns is crucial for developing strategies to promote equitable access to sport and physical activity across all age groups.

The financial commitment required for sport participation in Australia also varies across different age groups and exhibits notable differences between genders. According to data from the 2022–2023 financial year (6, 17, 18, 32, 33), the average annual costs paid to organisations or venues for children's sport participation were $899 for ages 0–4, $1,382 for ages 5–8, $1,450 for ages 9–11, and $1,760 for ages 12–14 (31). These figures indicate a progressive increase in costs as children grow older, which can be attributed to factors such as higher competition levels, increased training and playing frequency, and the need for more specialised or expensive equipment. The lowest average cost is observed in the 0–4 age group, where participation often involves introductory programs with minimal fees. For children aged 5–9, many sports offer modified programs designed as entry-level experiences, which typically incur higher costs due to structured coaching and organised sessions (5).

Gender disparities in costs are also evident. For children aged 0–11, parents and carers paid more on average for female participants compared to males. This trend reverses in the 12–14 age bracket, where costs for male children surpass those for females. These differences may result from varying participation rates, the types of sports chosen, and the associated costs of equipment and training for each gender (34).

Among adults, the average annual cost to participate in sport during the 2022–2023 financial year was slightly lower than that for children, averaging $1,304 (6, 12, 17). Costs varied across age groups, with Eime et al. (31) reporting average annual costs of $1,200 for ages 15–17, $1,100 for ages 18–24, $1,250 for ages 25–34, $1,460 for ages 35–44, $1,300 for ages 45–54, $1,150 for ages 55–64, and $1,000 for those aged 65 and over. These figures demonstrate that the highest average costs are incurred by adults aged 35–44, potentially reflecting peak engagement in the most expensive organised sports and related activities such as golf. Notably, across all adult age groups, females consistently incurred slightly higher average costs than males. This disparity could be influenced by factors such as the types of sports participated in, the frequency of participation, and additional expenses related to equipment and training. Understanding these cost variations is crucial for developing targeted strategies to reduce financial barriers and promote inclusive participation in community sports across all demographics.

Over the five-year period from 2018 to 19 to 2022–23, the average annual cost of participating in organised sport in Australia has risen across various age groups (6, 12, 17). This upward trend reflects broader economic factors, including inflation and increased operational costs for sporting organisations. Among children aged 0–14 years, costs increased across all subgroups: for those aged 0–4, the average cost rose by $313 to reach $899; children aged 5–8 saw a rise of $587, reaching $1,382; for the 9–11 age group, costs grew by $436 to $1,450; and among 12–14 year-olds, the most significant increase of $583 pushed the average annual cost to $1,760. On average, children's sport participation costs escalated by $487 over this period. Factors contributing to this rise include higher fees for structured programs, increased prices for equipment and uniforms, and additional expenses associated with play at a higher level of (sub-elite) competition. These financial demands can pose challenges for families, potentially impacting children's continued involvement in organised sports.

Adults also experienced rising participation costs during this period. The average annual cost for adults aged 25–34 increased by $489, while those aged 55–64 saw a similar rise of $486. Overall, adults experienced an average cost increase of $391. This trend may be linked to heightened participation in fitness and gym activities, which often involve membership fees and additional costs for specialised classes or personal training sessions (21).

## Informal and low-cost participation

Much of the evidence on costs derives from organised, club-based sport. Yet a growing body of research demonstrates that informal sport and active recreation such as pick-up basketball, casual football in parks, skateboarding, running, and other non-club settings make a substantial contribution to overall physical activity (14, 35). These forms of participation are typically low-cost, relying on free or publicly available spaces, and are often more flexible and inclusive for groups that face barriers to structured sport. Importantly, informal participation can foster social connection and physical literacy without the recurring financial burdens of registration, uniforms, or travel. However, because policy analyses tend to focus on organised sport, the cost advantages and participation potential of informal sport remain under-recognised in debates about affordability.

## Cost to community clubs to deliver sport

In addition to the rising costs faced by participants, community sport clubs themselves are grappling with mounting financial pressures that jeopardise their ability to deliver affordable and sustainable sport programs and competitions. A recent industry report highlights that community sports clubs in Australia are encountering escalating financial pressures that threaten their sustainability. A 2023 survey by the Australian Sports Foundation revealed that rising operational costs and declining revenues have pushed nearly one in five (18%) community sporting clubs to the brink of collapse (12).These challenges are compounded by increased competition from commercial sports providers, which often employ professional staff, thereby reducing volunteer involvement and imposing additional financial burdens on non-profit clubs. There is some evidence about key expenses that are contributing to the financial strain experienced by community sports clubs. These costs are first of all facility expenses—the construction and maintenance of sports facilities represent substantial costs. Internationally, clubs that own their facilities face significant development and upkeep expenses, while those utilising public venues may encounter rising rental fees. Next are insurance costs—escalating insurance premiums have become a critical issue, with sport and recreation organisations disproportionately affected by increases in public liability insurance premiums (36). Staffing costs are increasing because of the growing need to replace volunteers with paid staff. This adds further strain to already tight budgets. And finally there are the environmental costs of natural disasters, such as droughts, fires, and floods, that pose financial risks to local sports clubs, as they can cause damage to facilities and disrupt operations (36).

In New Zealand, the sport sector has experienced significant revenue declines in recent years, particularly from club memberships and sponsorships, leading to sustained financial pressure on community sports organisations (37). In Australia, but also in other countries, governments and sport governing bodies have engaged in different strategies to lift the cost burden on participants.

## Strategies to improve affordability and sustainability

To mitigate financial pressures and enhance the affordability of community sports, several strategies have been utilised. Australian government initiatives, such as voucher programs, provide direct financial assistance to families, encouraging children's participation in sports (34). Within the sport clubs, leveraging volunteers for administrative tasks, coaching, and event management can significantly reduce labour costs while fostering community involvement. Beyond better using the almost free labour from volunteers, cost-saving measures, such as reducing energy consumption and deferring non-essential maintenance, can also help clubs manage expenses during financially challenging periods (12). Increasingly necessitated by the ever rising costs of delivering sport participation opportunities, clubs and their governing bodies are exploring alternative revenue sources, including hosting community events, offering exclusive membership packages, and selling merchandise online. This can diversify income and enhance financial stability (12).

## Direct financial incentives—vouchers

To mitigate financial barriers hindering children's participation in organised sport, both Australian and international governments have increasingly implemented sports voucher programs. These initiatives aim to alleviate costs associated with sports participation, thereby promoting physical activity among children and youth.

In Australia, sports voucher programs have been adopted across multiple states and territories, including South Australia, Victoria, New South Wales (NSW), Queensland, Western Australia, Tasmania, and the Northern Territory. Since 2011, these programs have operated without national standardisation, leading to diverse approaches tailored to regional needs (34). The variation in eligible community access to vouchers across regions ranged from 80% in the Northern Territory to 11% in South Australia, underscoring the importance of targeting these programs to support disadvantaged populations effectively (38).

The uptake of sports vouchers among eligible recipients in Australia varies between 46% and 53% across state programs (38). However, lower socio-economic status (SES) families and those in regional areas often exhibit reduced awareness or access to these programs compared to higher SES families. This disparity highlights existing inequalities in the awareness and accessibility of financial incentive programs, potentially limiting their effectiveness (20, 34, 39).

Universal financial voucher offers are generally easy to administer and have a broad reach. However, evidence suggests that such schemes may disproportionately benefit higher SES groups, thereby exacerbating existing inequalities (34). Targeted, means-tested programs offer financial assistance to those in greatest need, potentially allowing for higher-value vouchers to fewer recipients. While some Australian states have implemented targeted programs, there is limited published data on their uptake and effectiveness. Ensuring equity of access is crucial to avoid widening socio-economic disparities (34).

## Challenges and considerations

Targeted approaches present challenges, including the risk of stigmatisation and increased administrative complexity, which can create barriers for participants and providers. For families in less disadvantaged circumstances, voucher schemes often help cover existing costs rather than creating new opportunities, functioning more as a bonus than essential financial aid (40). Additionally, there have been anecdotal reports of junior sports clubs raising fees following the introduction of voucher programs, potentially undermining the intended financial relief (34).

## Gender disparities and impact on physical activity levels

Financial incentive programs also have the potential to contribute to gender inequities, with boys more likely than girls to utilise opportunities for more affordable participation in competitive sport. In the NSW and South Australia voucher programs, boys were more likely to register and use a sports voucher (39, 41). However, in NSW, girls who did register and used a voucher reported greater increases in physical activity levels than boys, reducing the gender gap in overall physical activity levels (41).

Several studies have reported that voucher programs result in increased self-reported physical activity levels among children, with these increases maintained over a six-month period (34, 41). However, some parents have expressed concerns that beyond the one-off voucher payment, their child's long-term participation in the sport may be limited (40). Additionally, a German study involving 33,000 sports club membership vouchers found no significant short-term or long-term effects on sports club membership or physical activity levels, suggesting that vouchers alone may not be sufficient to overcome barriers to sustained participation (42).

## Indirect financial incentives

Indirect incentives, such as lotteries, tax rebates, and prize-based rewards, have been employed internationally to encourage physical activity and sports participation. These incentives operate by rewarding positive behaviours, thereby aiming to reduce financial barriers and promote active lifestyles (34). For instance, in Singapore, a study offered children incentives valued at US$20 if they met a daily step count target of 8,000 steps for at least half of the days in a month. While this approach led to short-term increases in physical activity, the improvements were not sustained beyond a six-month period (43).

There is evidence suggesting that such indirect financial incentives can undermine intrinsic motivation. Self-determination theory posits that external rewards for activities that are inherently interesting may diminish an individual's internal drive to engage in those activities once the incentives are removed (44). This phenomenon, known as the “crowding out” effect, indicates that while financial incentives can provide immediate motivation, they may negatively impact long-term adherence to physical activity (44). In the context of sports participation, intrinsic motivation—driven by factors like enjoyment and personal satisfaction—is crucial for sustained engagement (31).

Moreover, the design of incentive programs plays a significant role in effectiveness. Programs that offer immediate rewards, such as cash prizes or lotteries, may capitalise on present bias, providing an immediate payoff that contrasts with the delayed health benefits of exercise (44). However, the long-term sustainability of such programs remains questionable, as the removal of incentives often leads to a decline in the desired behaviour (43). While indirect financial incentives can temporarily boost physical activity levels, their potential to undermine intrinsic motivation and the lack of sustained behavioural change highlight the need for carefully designed programs. Strategies that balance extrinsic rewards with the promotion of intrinsic motivation may offer more effective and enduring solutions to increasing sports participation.

Tax rebates have been implemented in various countries as indirect financial incentives to promote children's participation in organised physical activity. For instance, Canada introduced the Children's Fitness Tax Credit (CFTC) to provide tax refunds for registration fees associated with organised physical activity programs (45). However, the effectiveness of such tax credits in increasing sport participation and overall physical activity levels among children has been limited. Research indicates that the CFTC did not lead to a significant increase in children's physical activity levels or improve self-reported health outcomes (46).

Furthermore, disparities in the utilisation of tax credits have been observed across different income groups. Higher-income families were more likely to be aware of and claim the CFTC compared to lower-income families. This discrepancy suggests that tax credits may inadvertently benefit those who are already financially capable of enrolling their children in organised activities, thereby widening the existing participation gap (45). Lower-income families often face immediate financial constraints that prevent them from affording the upfront costs of enrolling their children in sport and physical activity programs, making deferred financial incentives like tax credits less effective for these populations (47).

Similarly, a study in the United States investigated the impact of a simulated refundable tax credit on low-income children's participation in after-school physical activity programs. The findings revealed that the tax credit did not significantly influence enrolment rates, frequency of participation, or overall moderate-to-vigorous physical activity levels among the children (47). These outcomes highlight that the prospect of future tax reductions may not serve as a sufficient incentive to alter current behaviours related to sport and physical activity. Direct financial assistance to program providers for enrolling low-income children might be a more effective approach to increasing participation rates (47).

Sport lottery systems are another example of indirect financial incentives. They are utilised globally to generate funding for sports initiatives by allocating a portion of lottery ticket sales towards prizes and distributing the remaining funds to sports organisations and related causes. These systems operate at various administrative levels, including national, state, or provincial. The United Kingdom's National Lottery, established in 1994, has significantly impacted sports funding. Over three decades, it has raised over £48 billion for various causes, supporting more than 685,000 initiatives, including substantial investments in community sports and facilities. This funding model has been instrumental in enhancing sports infrastructure and promoting active lifestyles across the UK. It should be noted that the means of attracting such funds, principally through gambling activities, is in itself quite controversial. Especially in light of the increasing focus on attracting younger audiences to sports gambling platforms (48–51).

Similarly, in Ireland, the National Lottery was established in 1986 to support sport and recreation, national culture, the arts, and community health. The proceeds from the lottery have been instrumental in funding various sports projects and initiatives, thereby promoting physical activity and community engagement (52). In Australia, the concept of a national sports lottery has been proposed and debated on multiple occasions (19). In 2018, the Play for Purpose charity raffle was launched, supporting a range of sports organisations, including the Australian Sports Foundation and local clubs. Play for Purpose operates as a not-for-profit raffle, with each ticket contributing directly to participating charities and sporting clubs, thereby providing them with a revenue stream to support their activities (53).

Despite these initiatives, Australia does not have a unified national sports lottery system. Lotteries are regulated at the state level, leading to variations in implementation and funding distribution across different regions. Discussions in the 1980s about establishing a national lottery concluded that sport and recreation were not prioritised over other areas such as health and welfare. Concerns included the potential for funding to be driven by lottery subscriptions rather than the actual needs of the sporting community, the possibility of sport receiving preferential treatment over other sectors, and risks of inefficient spending within the sports portfolio. The idea resurfaced in the lead-up to the Sydney 2000 Olympics and Paralympics. However, uncertainties about whether such a lottery could attract additional funding, given the existing array of gambling options available to the public, hindered its implementation.

## Grant programs

In the 2023–24 financial year, the ASC launched the Play Well Participation Grant Program (54), allocating a total of $10.3 million to eligible organisations. The program's objectives are to increase involvement in sport and physical activity through inclusive and quality experiences and to address barriers preventing participation, providing more opportunities for those facing the most challenges (6).

Grants ranging from $10,000 to $300,000 are available to national sporting organisations, national sporting organisations for people with disabilities, national physical activity providers, and local government councils. While clubs are not eligible to apply directly, they are encouraged to collaborate with their state and national bodies to express interest in the program (6).

Previously, in the 2019–20 financial year, the ASC invested $56 million into two funding streams under the Move It AUS initiative (16). This included Participation Grants aimed at engaging Australians of all ages in organised sport and physical activity, and Better Ageing Grants, focused on encouraging those aged over 65 to become more active (15, 55).

A pragmatic, mixed-methods evaluation of these programs indicated success in engaging physically inactive participants. Key insights from qualitative interviews highlighted the importance of clarity in target demographics, effective partnerships, communication, program delivery, environmental impacts, governance, and prioritising physical inactivity. Despite challenges posed by the COVID-19 pandemic, the grant programs demonstrated that organised sport could effectively reach inactive populations, achieving positive health outcomes.

At the state and territory level, governments offer various financial support programs targeting participation or infrastructure development. These grants often focus on participation initiatives, addressing barriers to involvement in sport. They also include infrastructure support, funding the development or enhancement of sports facilities and capacity building whereby the focus is on enhancing the (human) capabilities within sport organisations.

For instance, the New South Wales Government's Local Sport Grant Program aims to increase regular participation opportunities in sport. In the 2023–24 cycle, up to $4.65 million was available, supporting projects such as purchasing uniforms and equipment, first-aid or safety training, and sports facility development (33).

## Fundraising and charity organisations

The Australian Sports Foundation (ASF) is a national non-profit organisation dedicated to fundraising for sports initiatives across Australia. Notably, it is the only organisation in the country where donations to sport are tax-deductible. In the 2023–24 financial year, the ASF achieved a record-breaking milestone by raising $98.8 million AUD from over 70,000 donations, significantly supporting community clubs and athletes nationwide (56, 57).

The ASF's efforts have been recognised in strategic discussions about enhancing community participation in sport (58). The “Future of Sport” report highlighted opportunities for corporations to channel donations through the ASF to benefit grassroots sports. It also recommended increased promotion of the ASF as a viable vehicle for raising funds dedicated to sporting activities (59). Beyond the ASF, several other not-for-profit organisations and charities work tirelessly to minimise the costs associated with sports participation, particularly focusing on disadvantaged groups.

One such organisation is Pass to Me, which provides new and used sporting equipment to disadvantaged youth. By sourcing equipment from schools, manufacturers, sporting clubs, and individual donors, Pass to Me aims to facilitate access to sport among disadvantaged youth, promoting benefits such as enhanced physical and mental well-being, improved social inclusion, and a reduction in rates of preventable diseases (60). Similarly, Kit Bag for Kids collects and distributes second-hand, surplus, or unwanted sporting goods to underprivileged children across Australia. This initiative not only provides essential equipment but also encourages school attendance by integrating sports participation with educational engagement (61).

Several retail companies have also established programs to support sports participation. For instance, Nike offers an in-store recycling and donation program for sporting footwear and apparel. They assess donated items to determine eligibility, clean usable gear for donation, and recycle products that cannot be reused. Notably, Nike accepts gently worn items from any brand, not just their own (62). Asics has partnered with Give Back Box to create a recycling program that operates both in-store and online. Customers can print a pre-paid label and reuse shipping boxes to return sporting goods, facilitating an easy and eco-friendly donation process (63). Another example is Rebel Sport, which supports grassroots participation and local communities through its Community Givebacks program. This initiative allows store purchases to be linked with credit redemption for clubs. However, as of now, the program is on hold due to a review (64). Collectively, these organisations and programs play a crucial role in reducing financial barriers to sports participation, ensuring that more individuals, regardless of their socio-economic status, have the opportunity to engage in and benefit from sporting activities.

## (International) Government sport policy

Government policies play a pivotal role in shaping sports participation by implementing initiatives that address financial and infrastructural barriers, thereby promoting inclusivity and accessibility. In Flanders, Belgium, sports policy initiatives have been geared towards enhancing participation through financial support and infrastructure development. Local administrations place a strong emphasis on subsidising voluntary associations, with sports clubs estimated to receive €16.3 million in subsidies from local governments. These subsidies aim to make sports more accessible, particularly for individuals from lower socio-economic backgrounds who may face challenges engaging in commercial sports environments. By focusing on community-based sports settings, these policies strive to accommodate diverse community needs and promote inclusivity (65, 66). 

In England, targeted initiatives have been introduced to support physical activity among lower socio-economic groups. For instance, the promotion of the Couch to Fitness free home workouts was funded to support people from lower socioeconomic backgrounds to continue to be active. This targeted marketing resulted in over 3,000 additional registrations, with 96% of participants residing in the most deprived areas (11). Globally, various forms of inequality—including economic, racial, geopolitical, and gender-based disparities—persist and impact participation in sport. While community sport has the potential to contribute to social justice, it requires a nuanced approach that differentiates between justice and charity. Darnell (67) argues that sport can contribute to positive social change when implemented with an understanding of social structures and cultural differences, moving beyond top-down approaches to include strong community engagement. However, he also warned against overstating the developmental and health potential of sport without acknowledging the political and economic structures that shape its delivery. Darnell (67) argues that sport-for-development and public health agendas can be compromised when they are embedded in neoliberal logics that prioritise market efficiency, corporate sponsorship, and individual responsibility. His analysis highlights the risks of policy reliance on corporate philanthropy or commercial branding, which may reproduce existing inequalities rather than dismantle them. Incorporating these insights underscores that while reducing the cost of participation is an important equity measure, sustainable progress requires structural safeguards to prevent community sport from being co-opted as a vehicle for commercial or political interests.

## Effectiveness of sport policies

A recent systematic review investigated the impact of sport policies on participation in sport and physical activity. The findings indicated that building sports facilities, reducing financial costs, and stimulating demand through sporting events have had some success in increasing sport participation, although the most global of them all, the Olympic Games, has shown no impact on long-term participation (68). Such policies have been more effective among individuals who were already moderately active and motivated, highlighting the “inequality paradox” where healthier individuals are more likely to engage in population-based interventions, potentially widening socioeconomic disparities (69). While these findings underline the limited effectiveness of large-scale events such as the Olympic Games in stimulating lasting participation, a broader body of scholarship suggests that the real question is not whether events inspire, but how they are strategically leveraged to generate sustainable community sport engagement. Further evidence consistently shows that inspiration generated by major events rarely results in a sustained “demonstration effect” without deliberate strategies to connect enthusiasm with accessible opportunities (70). A systematic review of the Olympic Games found little evidence of long-term grassroots participation growth, cautioning against relying on inspiration alone as a policy lever (70). More recent research highlights that intentional leveraging such as providing vouchers, structured pathways, or targeted club linkages, is required to translate event inspiration into actual participation (71–73). Event-related incentives can help overcome cost barriers and narrow the gap between intention and behaviour, particularly for volunteers and other highly engaged groups (72, 73). Collectively, this literature suggests that events of any scale can contribute to participation only when supported by equity-focused leveraging strategies, rather than assumed trickle-down benefits.

Additionally, policies focusing on sports clubs must consider the organisational resources and capacity to deliver targeted participation programs for underrepresented groups. Top-down approaches have often been unsuccessful due to factors such as authority, policy, and capacity constraints. Therefore, a broader systems approach is recommended, integrating the sports environment with other policies designed to promote physical activity. This aligns with the World Health Organisation's Global Action Plan for Physical Activity, which advocates for a comprehensive strategy to increase physical activity levels (74). Sport England (75) has adopted such a systems approach in their recent “Implementing Uniting the Movement 2022–2025” strategy, focusing specifically on tackling inequalities in sport and physical activity. This program is currently under evaluation to provide robust evidence on systems approaches to increase participation (11).

## Policy implications and future research

As briefly introduced earlier in this paper, while participation in community sport has been consistently linked to positive health and social outcomes, the evidence base also highlights important caveats. Not all sports confer uniform benefits, and risks must be carefully considered in policy design. For example, sport participation is associated with higher rates of acute injury and, in some codes, recurrent concussion and long-term musculoskeletal conditions such as osteoarthritis (76, 77). Mental health challenges, including disordered eating and body image pressures, have also been observed in certain athlete populations (77). These negative consequences do not negate the value of sport as a potential public health lever, but they underscore the importance of safe, inclusive, and well-governed environments that minimise harm while maximising health and equity gains. A policy agenda that positions sport as a contributor to public health must therefore also address injury prevention, coach education, safeguarding, and mental health support as integral components of participation systems.

The insights presented in this scoping review confirm that the rising costs of both participating in and delivering community sport pose significant challenges to equitable and sustainable sport systems in Australia. Financial barriers are particularly acute for lower socio-economic groups, culturally diverse populations, Indigenous communities, and people with disabilities, many of whom already face limited access to physical activity opportunities. At the same time, community sports clubs—often reliant on volunteers and inconsistent revenue—are experiencing growing financial stress due to increasing costs of facilities, insurance, staffing, and environmental disruptions. These dual pressures threaten the role of sport as a contributor to public health and social inclusion.

To address these challenges, future policy directions must reposition the affordability of sport as a public good and include discussion on the value of sport, rather than a market-driven service. National and state governments could embed sport affordability more explicitly into preventative health and wellbeing frameworks, recognising that equitable participation in sport contributes directly to physical, mental, and community health outcomes. Moreover, current funding models—which depend heavily on participant fees and voluntary labour—require recalibration. Standardised voucher systems, scaled according to socio-economic status, would offer more equitable access, while greater transparency and reform of affiliation fee structures could ensure that the financial burden borne by clubs and participants aligns with the actual value and services delivered.

In parallel, sustained investment in the organisational capacity of community clubs is essential. Many clubs lack the internal capability to pursue diversified revenue streams, navigate grant opportunities, or respond effectively to increased governance and compliance requirements. Policies that support upskilling volunteers, foster shared service arrangements across clubs and local governments, and facilitate access to tax-deductible fundraising platforms such as the Australian Sports Foundation can strengthen the resilience of grassroots sport organisations. Importantly, such support should be designed to reflect the realities of under-resourced communities, where administrative burden and digital capability remain persistent constraints.

Ensuring that sport policies deliver not just equal opportunity, but equitable outcomes, also demands more targeted strategies. While universal financial support schemes offer broad political appeal, they often benefit those already engaged in sport. In contrast, place-based, co-designed programs tailored to the needs of specific communities—whether different population groups like older adults or newly arrived migrant groups or indigenous communities—are more likely to produce meaningful shifts in participation. To avoid reinforcing existing inequities, policies must be assessed through a health equity lens, supported by comprehensive data collection and evaluation systems that can lesson the participation gaps across age, gender, ethnicity, geography, and ability.

The question of how to ethically fund sport remains a matter of ongoing debate. For example, national lotteries or gambling-linked revenues can contribute to a range of negative issues. If participation in sport is a human right, then we must find a way to make it more accessible.

While lottery revenues and corporate sponsorship have historically provided significant resources for community and elite sport, reliance on these mechanisms is not without risk. Lottery funding is often criticised as regressive, drawing disproportionately from lower-income households, while corporate sponsorship can embed commercial priorities and branding agendas within community sport systems (78, 79). To ensure that such funding models support equity rather than exacerbate inequalities, transparent governance, ring-fenced allocations for community participation, and safeguards against mission drift are essential.

Future research should address a number of pressing gaps. While a growing body of literature has assessed short-term outcomes of financial interventions such as voucher schemes, fewer studies examine their long-term effectiveness in sustaining participation over time, particularly for priority populations. Longitudinal designs could offer more robust evidence of how subsidies interact with other social determinants of participation, and whether drop-out rates rise once financial support is removed.

In addition, economic modelling studies are needed to more precisely estimate the return on investment in sport affordability initiatives. By comparing the upfront cost of subsidy programs or club grants with the downstream savings in health care, mental health services, and social inclusion, policymakers could build a stronger fiscal rationale for funding allocation. Similarly, better financial tracking of community clubs would help monitor the evolving cost base of sport delivery and identify early warning signs of organisational distress.

Policy frameworks that seek to improve affordability should also broaden their scope beyond club-based sport. Evidence suggests that informal participation not only attracts diverse cohorts but also provides accessible pathways for those who cannot afford structured programs (14, 35). Governments can support informal sport by ensuring the provision and maintenance of safe, well-lit, and freely accessible facilities such as parks, schoolyards, and community courts. Policies that enable shared-use agreements between schools, councils, and community groups, and that promote flexible access to space, may deliver significant physical activity benefits at relatively low cost.

Research should also look beyond financial variables alone. Cost is rarely the sole determinant of sport participation. Further inquiry is needed into how financial, cultural, motivational, and logistical barriers interact—particularly in relation to migrant, Indigenous, and disabled populations. Finally, there is a growing need to understand the role of digital innovation in reshaping cost structures in community sport. As clubs begin to adopt AI-driven management systems, virtual training platforms, and online fundraising tools, evaluating the accessibility, effectiveness, and unintended consequences of such technologies becomes an important avenue for future inquiry. It can be argued that a stronger focus on identifying what is the value of sport is required which then can deliver more compelling evidence of the long-term health and wellbeing savings participation in community sport provides to the health system.

Our analysis suggests that affordability can indeed not be separated from governance. Viewing participation through the lens of (in)formalisation highlights how negotiations over facilities, scheduling, insurance and institutional recognition shape who can access low-cost and flexible options alongside club-based pathways. Accordingly, an equity-oriented agenda must pair targeted price relief with reforms that make these governing processes more transparent and inclusive so that both “formal” and “informal” opportunities are expanded without reproducing existing inequalities (14).

A new policy agenda is required—one that views sport participation not merely as a lifestyle choice but also as potential contributor to public health and social equity while ensuring policies also mitigate risks and avoid assuming uniform benefits across all forms of sport participation. Achieving this will demand sustained commitment to affordability, stronger support for delivery systems, and an ongoing research effort that informs policy design with precision, nuance, and community voice.
